# The History of Anæsthesia

**Published:** 1870-11

**Authors:** 


### The History of Anæsthesia.

Much has been said and written on this topic, and no small amount of angry controversy has for many years been expended upon it. A very decided and unfortunate impulse was given to the dispute by the unnecessary, as we have always considered it, attack on the late Prof. Simpson, by Dr. Bigelow, of Boston, and the Journals, both here and abroad, have rushed into the quarrel, taking one or other side, according as their information and predilections guided them. Although oceans of ink have been spilled in putting on record all these opposing opinions, we have nowhere seen the whole subject so neatly , and in our estimation justly and candidly, summed up, as by Dr. Bennett, the President of the Medico-Chirurgical Society of Edinburgh, in officially announcing to that body the death of Sir James Y. Simpson. Dr. Bennett was Secretary of the Society at the time of Simpson's investigations, which were first communicated to the Society. He was also at that time editor of the *Medical Journal,* and published therein a monthly record of the progress of etherization, and of the introduction of chloroform as a substitute for ether. He was also Chairman of the Committee appointed by the same Society to investigate the properties of chloroform, and in this official capacity he drew up the Chloroform Report, which was published in the *Edinburgh Journal,* January, 1848. His opportunities, therefore, for sifting the truth out of all this varied controversy have been unexceptionable, and we think he is entitled to speak with some degree of authority thereon. His views of the share of credit which properly belongs to the three American claimants accord precisely with those we have always maintained, and which we venture again to lay before our readers:

1. That numerous efforts in past times had been made and suggested for rendering persons insensible during pain, however produced. Physiologists had successfully used them when experimenting on the lower animals, and in medicine they were extensively employed. But all such efforts had been abandoned during surgical operations in man previous to 1844, as being either not practical or not safe.

2. In that year (1844), Dr. Horace Wells, a dentist, of Hartford, Connecticut, U. S., employed nitrous-oxide gas for extracting teeth, without pain, having, as he tells us in a pamphlet he subsequently published, been led to do so by observing that persons under the influence of that gas, or when greatly excited, as in battle, or from intoxication, were insensible to injuries inflicted upon them. It had, however, been recommended for this very purpose by Sir Humphrey Davy fifty years previously.

3. Dr. Wells communicated his ideas to the medical men of Boston, U. S., including Drs. Morton and Jackson, the former also a dentist, and a previous pupil and partner of his own. He endeavored to show the good effects of nitrous oxide gas, in the theatre of the Massachusetts General Hospital, but having withdrawn the bag containing it too soon, the experiment failed—a circumstance which disencouraged for two years public efforts in this direction.

4. The vapor of sulphuric ether was first employed by Dr. Morton, in Boston, U. S., on the 30th of September, 1845, in the successful extraction of a tooth, without pain; and shortly afterward it was also successfully used in surgical operations, and became generally employed in America and Europe during the following November and December. It would appear, however, that this was done, if not altogether by the suggestion, at least with the advice and encouragement of Dr. Jackson, who subsequently disputed with Morton the honor of the discovery.

5. On the 19th of January, 1847, ether was first employed in a difficult case of midwifery, by Sir James Simpson, in the presence of Dr. Ziegler, Dr. Keith, and Mr. Figg. An important obstetrical operation was performed, and it was ascertained that this, the progress of the labor, and its successful conclusion, were accomplished without pain, the consciousness of the patient having been suspended for a considerable time.

6. We are informed by Prof. Miller, that on the 4th of November, 1847, chloroform was first tried by Sir James Simpson on himself, and on his assistants, Drs. Keith and Duncan; and, in the first paper Sir James published on the subject, he tells us that the drug was recommended to him for that purpose by Mr. Waldie, of the Apothecaries' Company in Liverpool. On the 10th of that month, it was publicly made known at the evening meeting of this society. Many of the members inhaled it, and its agreeable odor, rapid and powerful effects as an anæsthetic, and general superiority to sulphuric ether, were at once admitted by those who tried it. The enthusiasm it created, and the remarkable aspect of a very crowded meeting, half of whom were temporarily excited and intoxicated by the drug, will not be readily forgotten by those who were present.

7. Up to this time the expressions etherization and narcotism had been correctly applied to the process and result of producing insensibility by means of these drugs, but Sir James Simpson now introduced the terms anæsthesia and anæsthetics, with a view of not alarming the public. It should be understood, however, that the effect is produced by suspending consciousness, and therefore sensation and volition, by acting on the brain and medulla oblongata in exactly the same manner, as is done by opium, alcohol, and other narcotics.

8. This is the more important to be attended to, as occasional deaths were soon reported as being caused by chloroform. These, it is true, are few, when compared with the many cases in which the drug was employed in dentistry, surgery, and midwifery, but have been in the aggregate sufficiently numerous to induce great caution, and a constant sense of anxiety among the profession at large. No fatal results, it is alleged, have been caused by the use of nitrous-oxide gas or sulphuric ether.

9. Other means of producing a true anæsthesia have been since sought after. Dr. James Arnot, of London, introduced powerful ice and frigorific mixtures, which, by freezing the part, admitted of operations being performed without pain. More recently, Dr. B. Richardson, of London, effected the same object, by pumping the spray of a pure ether on the part. It is to be observed, however, that the intense feeling of cold thus occasioned is disagreeable, often extremely painful, and that these methods, therefore, have never supplanted chloroform in dentistry and surgery.

10. During the last two years, nitrous oxide gas has again been introduced with great success in dentistry; its preparation and mode of administration having been much improved, principally through the efforts of Mr. Clover, of London. But it has not superseded chloroform in surgery, as it has not yet been considered safe to prolong sufficiently the effects it produces.

*Lastly.* Other narcotic agents have been employed, especially by Dr. Richardson, of London, to remove pain, such as the bichloride of methylene, the tetrachloride of carbon, the chloride and nitrate of amyle, with varying results. Only last year the hydrate of chloral was introduced as an hypnotic and anæsthetic, by Dr. Liebreich, of Berlin, and it is now being generally tried and investigated by the medical profession. None of these agents up to this time has superseded chloroform.

From this record, it appears to me, as regards the three great anæsthetics which have been largely used, the first, nitrous-oxide gas, was suggested by Sir Humphrey Davy, and introduced into dental practice by Dr. Horace Wells; the second, ether, was recommended by Dr. Jackson, and first introduced into dentistry and surgery by Dr. Morton, and into midwifery by Sir James Simpson; and the third, chloroform, was suggested by Mr. Waldie, and applied to medical practice generally by Sir James Simpson.—*New York Medical Journal.*

